# The optimization of cell therapy by combinational application with apicidin-treated mesenchymal stem cells after myocardial infarction

**DOI:** 10.18632/oncotarget.17471

**Published:** 2017-04-27

**Authors:** Dong Im Cho, Wan Seok Kang, Moon Hwa Hong, Hye Jin Kang, Mi Ra Kim, Min Chul Kim, Yong Sook Kim, Youngkeun Ahn

**Affiliations:** ^1^ Cell Regeneration Research Center, Chonnam National University Hospital, Gwangju, South Korea; ^2^ Department of Cardiology, Chonnam National University Hospital, Gwangju, South Korea; ^3^ Biomedical Research Institute, Chonnam National University Hospital, Gwangju, South Korea

**Keywords:** mesenchymal stem cell, myocardial infarction, apicidin, differentiation, cotransplantation

## Abstract

Although mesenchymal stem cells (MSC) have been shown to be safe in preclinical studies of cardiovascular disease, multiple meta-analyses have debated whether functional improvement is significant or not. The cardiac differentiation from MSC is achievable using cardiogenic factors, however, the high cost and long culture period may limit the applications. Here, we developed a novel method to optimize the therapeutic outcome for myocardial infarction (MI). Treatment of MSC with apicidin, a histone deacetylase inhibitor, dramatically increased the expressions of cardiac markers such as GATA4, Nkx2.5, and cardiac troponin I (cTnI). In AC/MSC, stemness-related genes and yes-associated protein (YAP), a potent oncogene that drives cell proliferation, were significantly suppressed. Furthermore apicidin treatment or YAP knockdown downregulated miR-130a expression followed by induction of cardiac markers in MSC. In the comparison study, we found that both cardiac gene induction and angiogenesis were most prominent in the mixture of non-treated MSC and AC/MSC (Mix). Using mouse MI model, we show that application of Mix was strongly associated with cardiac differentiation of injected MSC and improved cardiac performance. Our results suggest that suppression of YAP/miR-130a shifts MSC cell fate toward cardiac lineage and identify apicidin as a potential pharmacological target for therapeutic development.

## INTRODUCTION

Mesenchymal stem cells (MSC) are regarded as safe and feasible for cardiovascular therapy in clinical applications [1–3]. Ventricular function is significantly improved after stem cell therapy, mainly in association with the induction of angiogenesis [4], the paracrine effect [5], or stimulation of endogenous cardiac progenitor cells [6]. Despite intense efforts, however, recent multiple meta-analyses have debated whether the therapeutic efficacy of MSC treatment is significant [7, 8].

Debate remains as to whether differentiation into mature cardiomyocytes would be better than the application of undifferentiated stem cells to damaged heart. Induction of cardiac-specific gene Nkx2.5 expression by genetic modification in P19 cells appears to be associated with enhanced cardiac differentiation [9]. Enforced expression of GATA4, Mef2c, and Tbx5 by using retroviral vectors in fibroblasts showed reprogramming cell lineage into cardiomyocytes [10]. This study raised the possibility to directly transdifferentiate into functional cardiomyocytes in the scar tissue after cardiac injury. In terms of cardiac differentiation efficiency, the process of reprogramming fibroblasts to cardiomyocytes reported to be insufficient for clinical application [11]. Besides, concerns about the safety of viral vector, perfect purification of differentiated cardiomyocytes from undifferentiated cells, and time-consuming protocols are still remained unsolved.

Thus, intense investigation has focused on inducing cardiomyogenic differentiation, because adult stem cells such as MSC show poor efficiency and require several weeks to be cardiomyogenic. Despite this long-standing controversy over what type of cells can optimize cardiac regeneration, recent studies have shown that a modification or priming of the stem cells can lead to significant cardiac repair in animals. Treatment of stem cells that predisposes the cells to differentiate to cardiac cells has been described to have enhanced therapeutic efficacy in animal studies. In 1999, 5-Azacytidine was shown to induce cardiac differentiation in mouse bone marrow-derived MSC, and despite extremely low efficiency, this was the first report to show the possibility of cardiac differentiation of MSC [12]. Treatment of P19 embryonic carcinoma stem cells with trichostatin A was shown to induce differentiation into the cardiac muscle lineage [13]. 5-Azacytidine-treated human adipose tissue-derived stem cells failed to differentiate into cardiomyocytes, whereas trichostatin A treatment for up to 3 weeks or co-culture with neonatal rat cardiomyocytes increased differentiation into cardiomyocytes [14]. Rat bone marrow-derived MSC showed cardiac differentiation after 2 weeks of sequential treatment with 5-azacytidine, tricostatin A, and co-culture with neonatal cardiomyocytes [15]. Priming of umbilical cord blood-derived MSC with oxytocin for 7 days induces cardiac differentiation in the infarcted myocardium, which results in improved cardiac recovery [5]. Treatment of MSC with growth factor cocktails also improves the therapeutic effect for cardiac repair [16]. Recently, cardiogenically oriented MSC therapy was shown to have benefit in chronic heart failure in animal study [17] and the C-CURE clinical study [18]. They treated human bone marrow-derived MSC with cardiogenic growth factors containing a platelet lysate, transforming growth factor-β, bone morphogenetic protein 4, activin-A, fibroblast growth factor 2, α-thrombin, and cardiotrophin for 5 days for endomyocardial injection to ischemic heart failure patients. Cell therapy by using MSC with cardiogenic cocktail-based priming showed improved ventricular function and physical performance and quality of life.

Considering that stem cells are largely involved in cardiac regeneration and structural recovery after cardiac injury, we aimed to overcome the limitations and shortcomings of a singular approach for cardiac differentiation of MSC.

In this study, we found that apicidin induces cardiac commitment of MSC. Apicidin is an inhibitor of histone deacetylase (HDAC) and results in a dramatic increase of cardiac genes in MSC within 24 hours of treatment. The most of known protocols described above needed up to 4 weeks in epigenetic modulator-treated MSC. On the other hand, our protocol has the shortest induction time for cardiac markers.

Prior to differentiation, most mammalian cells arrest their growth at a state in the G1 phase of the cell cycle [19]. In human pluripotent cells, regulation of cell proliferation is mediated by the coupling of growth arrest and differentiation [20], and cell fate decisions are regulated by the cell-cycle machinery [21]. Yes-associated protein (YAP) is a major downstream effector of the Hippo signaling cascade that controls cell proliferation and organ size [22]. In embryonic stem cells, YAP is a transcriptional coactivator that modulates the pluripotency [23]. In this study, apicidin treatment drastically downregulated both mRNA and protein expression of YAP in MSC, and we suggested that the suppression of pro-proliferative mediators provides a remarkably efficient and rapid approach to transdifferentiation of stem cells. In the current study, we have assessed whether cardiac-committed MSC can improve therapeutic efficacy, and developed a novel protocol to maximize the therapeutic efficacy.

## RESULTS

### Cardiac markers are specifically induced in AC/MSC

**Figure 1:**
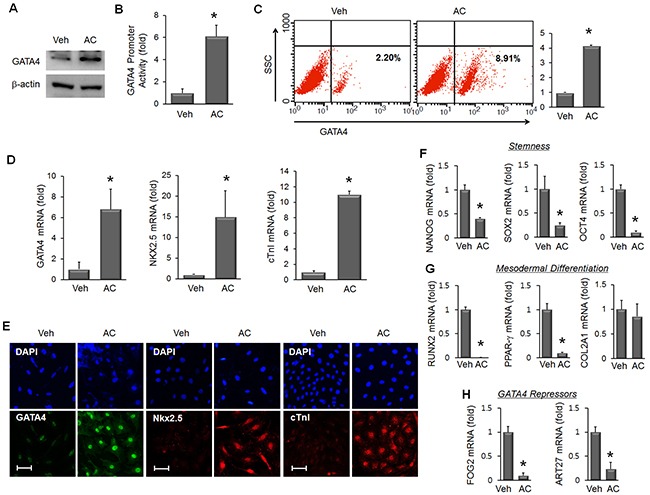
Effects of apicidin on inductions of cardiac genes, stemness, and differentiation capacity in bone marrow-derived mesenchymal stem cells (MSC) **(A)** MSC were treated with vehicle (Veh) or apicidin (AC, 3 μM) for 24 hours, and the protein level of GATA4 was assessed by Western blot. **(B)** GATA4 promoter activity was increased by TSA and apicidin treatment (n=6). **(C)** GATA4 protein was assessed by flow cytometry and the relative GATA4 expression level was expressed as a graph (n=4). **(D)** mRNA levels of GATA4, Nkx2.5, and cTnI were assessed by real-time PCR in vehicle-treated or AC/MSC (n=6). **(E)** Protein expression of GATA4, Nkx2.5, and cTnI was assessed by immunofluorescence staining. Scale bar=50 μm. **(F)** mRNA expression of the stemness markers Nanog, Sox2, and Oct4 was reduced in MSCs by apicidin (3 μM) treatment (n=5). **(G)** mRNA expression of the osteogenic marker Runx2 and adipogenic marker PPAR-γ was reduced, whereas that of the chondrogenic marker Col2a1 was not changed by apicidin treatment (n=5). **(H)** mRNA expression of FOG2 and ART27, transcriptional inhibitors of GATA4, was significantly reduced by apicidin treatment. Veh, vehicle. n=5, *P<0.05.

MSC were treated with vehicle (Veh) or apicidin (AC, 3 μM) for 24 hours to analyze the induction of cardiac markers. GATA4 protein was significantly increased only in AC/MSC (Figure 1A), and GATA4 promoter activity was increased by apicidin (Figure 1B). Flow cytometric analysis also showed an increase of GATA4 protein in AC/MSC (Figure 1C). To examine whether apicidin induced cardiac markers above GATA4, we additionally tested the expressions of Nkx2.5 and cTnI. The levels of mRNA (Figure 1D) and protein (Figure 1E) of GATA4, Nkx2.5, and cTnI were significantly increased in AC/MSC. Apicidin markedly inhibited HDAC activity of MSC (Supplementary Figure 1A). In AC/MSC, acetylated histone 3 and histone 4 were remarkably increased (Supplementary Figure 1B).

### Cell characteristics are altered in AC/ MSC

To examine the effect of apicidin on the stemness of MSC, we assessed the gene expression of Nanog, Sox2, and Oct4. The expression of these genes was significantly reduced by apicidin treatment (Figure 1F). To gain insights into the mechanism of cardiac lineage specification, we examined the effect of apicidin on the differentiation capacity of MSC. With apicidin treatment, expression of the osteogenic differentiation marker Runx2 and the adipogenic marker PPAR-γ were dramatically reduced, whereas that of the chondrogenic differentiation marker Col2a1 remained unchanged (Figure 1G). Simultaneously, apicidin represses genes inducing adipogenic, osteogenic, and chondrogenic differentiation. Meanwhile, endogenous inhibitors of GATA4, Fog2 [24], and ART27 [25] were also significantly decreased by apicidin treatment (Figure 1H). These data suggest that the effects of AC/MSC include a loss of stemness, reduced differentiation capacity, and downregulation of GATA4 inhibitors.

### The therapeutic efficacy of AC/MSC in a myocardial infarction model

**Figure 2:**
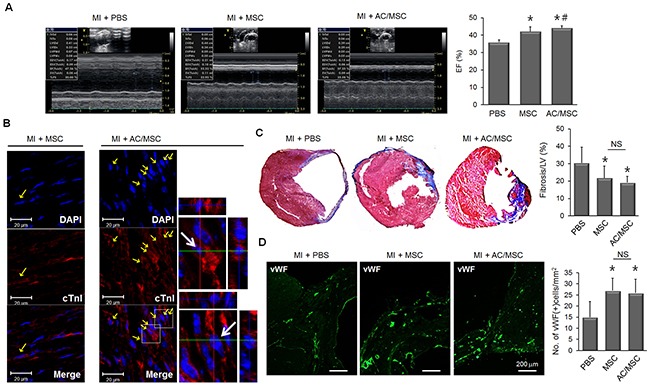
Therapeutic effect of AC/MSC MSC were injected into the peri-infarct zone at 7 days after induction of MI by coronary artery ligation. At 2 weeks after MSC injection, cardiac function was assessed by echocardiography, and heart tissue was isolated for histological studies. Before injection, MSC were labeled with DAPI for identification in the myocardium. **(A)** Representative M-mode images and EF (%) of the PBS group (n=10), the MSC group (n=8), and the AC/MSC group (n=12) are shown. **(B)** The expression of cTnI in the engrafted MSC was detected by immunofluorescence staining. Scale bar=20 μm. **(C)** Cardiac fibrosis was assayed by Masson's trichrome staining, and fibrotic area was quantified. **(D)** Angiogenesis in the peri-infarct zone was assessed by staining with vWF and the number of vWF-positive cells was counted. Scale bar=200 μm.

Next, we assessed the therapeutic effect of MSC in a mouse MI model. PBS, MSC, or AC/MSC were injected into the peri-infarct zone 1 week after MI. At 2 weeks after cell injection, echocardiographic analysis showed that ejection fraction (EF) was improved in both the MSC group and the AC/MSC group with statistical significance (Figure 2A, Supplementary Table 1). Double fluorescence images for cTnI (red) and DAPI-labeled MSC (blue) suggested that injected MSC differentiated to cardiac lineage cells. Cardiac differentiation was higher in the AC/MSC group than in the MSC group, which was consistent with the *in vitro* data (Figure 2B). Both cardiac fibrosis (Figure 2C) and angiogenesis (Figure 2D) were improved in the MSC group and AC/MSC group, but did not differ significantly between the MSC group and the AC/MSC group. Although more increase of cardiac differentiation of injected AC/MSC, the improvement of EF was marginal, cardiac fibrosis and angiogenesis did not show statistical differences between the MSC group and the AC/MSC group.

### Angiogenic activity is restored by MSC combination

**Figure 3:**
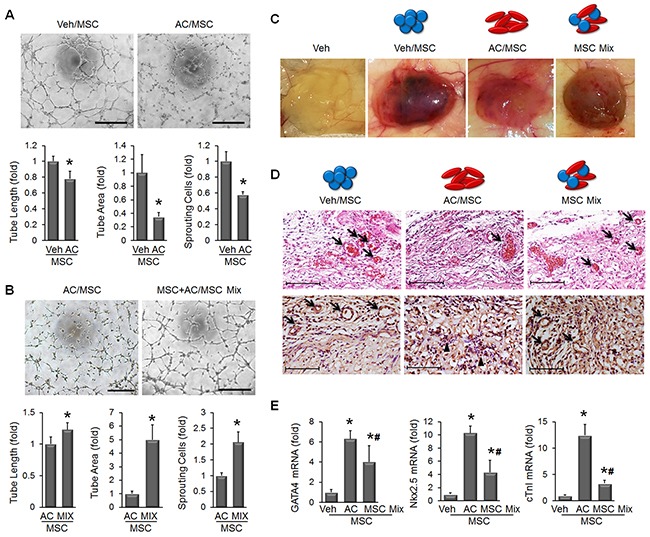
Combination of MSC and AC/MSC restores angiogenic activity **(A)** The representative images showed that tube formation was declined in AC/MSC. Tube length, tube area, and the number of sprouting cells were quantified as graphs in lower panel. n=5, Scale bar=200 μm. **(B)** The decline in tube formation in AC/MSC was significantly recovered by mixing with MSC. n=5, Scale bar=200 μm. **(C)** Plug assay was performed by subcutaneous implantation of MSC containing Matrigel into nude mouse. One week later, implanted plugs were harvested (n=4). **(D)** In H&E stained plugs, red blood cells containing vascular structures were decreased in the AC/MSC but while recovered in the MSC Mix group (upper panels). CD31(+) capillaries were also decreased in the AC/MSC group but restored in the MSC Mix group (lower panels). Scale bar=100 μm **(E)** mRNA levels of cardiac markers were decreased but still remained upregulated in harvested plugs that had been injected with the MSC Mix. n=4, *P<0.05.

Stem cell-induced angiogenesis is well known to contribute to tissue regeneration in ischemic lesions, and we examined whether apicidin treatment influenced angiogenic activity of MSC by quantifications of angiogenesis activity-related parameters such as tube length, tube area and sprouting cells. We found that AC/MSC showed a decline in the angiogenesis capacity (Figure 3A). To restore tube formation activity, we developed a novel protocol in which we mixed MSC and AC/MSC to compensate for the decline in angiogenic activity. We designated this as MSC Mix. Tube formation was substantially disturbed in AC/MSC, but was almost completely recovered in MSC Mix (Figure 3B). To determine whether MSC are involved in functional vessel formation, the plug assay was performed. Gross images showed retarded angiogenesis in plugs injected with AC/MSC and greater angiogenesis in plugs injected with the MSC Mix (Figure 3C). H&E staining also demonstrated red blood cells containing vascular structures in the MSC Mix group (Figure 3D, upper panels) and more CD31-positive capillary vessels (Figure 3D, lower panels). In terms of apicidin-induced cardiac markers, mRNA level of GATA4, Nkx2.5, and cTnI in MSC Mix was lower than in AC/MSC, but still remained significantly upregulated (Figure 3E).

### Optimized therapeutic efficacy of MSC Mix in a myocardial infarction model

**Figure 4:**
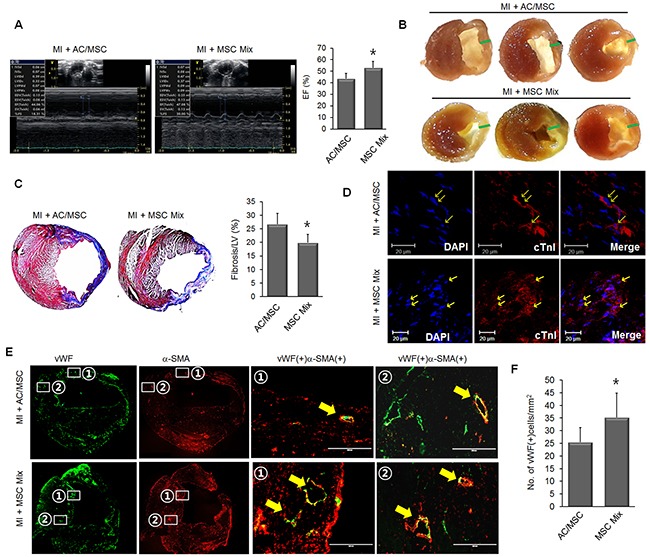
Enhanced therapeutic efficacy of MSC mix **(A)** Representative M-mode images of the AC/MSC group (n=8) and the MSC Mix group (n=8) are shown. **(B)** Gross images of the heart section from the AC/MSC group and MSC Mix group are shown and green bars indicated the wall thickness. **(C)** Cardiac fibrosis was assayed by Masson's trichrome staining, and fibrotic area was quantified. **(D)** The expression of cTnI in the engrafted MSC was detected by immunofluorescence staining. Scale bar=20 μm. **(E)** Induction of neovascularization was assessed by immunohistochemical staining, and these results demonstrated significantly greater numbers of capillaries and arterioles (defined, respectively, by vWF staining alone and combined expression of vWF and anti-SMA) in the MSC Mix group than in the AC/MSC group. vWF/α-SMA double-positive vessel structures (yellow arrows) were shown in the magnified images. **(F)** Angiogenesis in the peri-infarc zone was assessed by staining with vWF and the number of vWF-positive cells was counted. Scale bar=200 μm.

Next, we compared the therapeutic effect between AC/MSC and MSC Mix in a mouse MI model. Improvement of EF was greater in the MSC Mix group than in the AC/MSC group (Figure 4A, Supplementary Table 2). Representative images of cross sections of heart tissue also showed that ventricular wall thickness was better preserved in the MSC Mix group than in the AC/MSC group (Figure 4B). Masson's trichrome staining also showed that cardiac fibrosis was significantly alleviated in the MSC Mix group compared with the AC/MSC group (Figure 4C). The frequency of cardiac differentiation of injected MSC was similar both in the AC/MSC group the MSC Mix group (Figure 4D). In order to evaluate the effect of MSC application on the vascularity, we performed immunohistochemical staining with vWF and α-SMA in heart tissues 2 weeks post-MI. Consistent with better EF (%), there was a significantly higher vascular density in the MSC mix group. Furthermore, magnified images from white boxes were shown in merged images, and the newly formed microvessels in the MSC Mix group were more matured microvasculature accompanied by α-SMA as compared to the AC/MSC group (Figure 4E). In the MSC Mix group, marked induction of angiogenesis was observed in the MSC Mix group, as defined by greater numbers of vessels staining with endothelial marker vWF (Figure 4F). These results demonstrated that MSC Mix preserved both cardiac commitment and angiogenic activity enough for enhanced cardiac repair.

### Cell cycle regulators and GSK3β/ β-catenin are altered in AC/MSC

**Figure 5:**
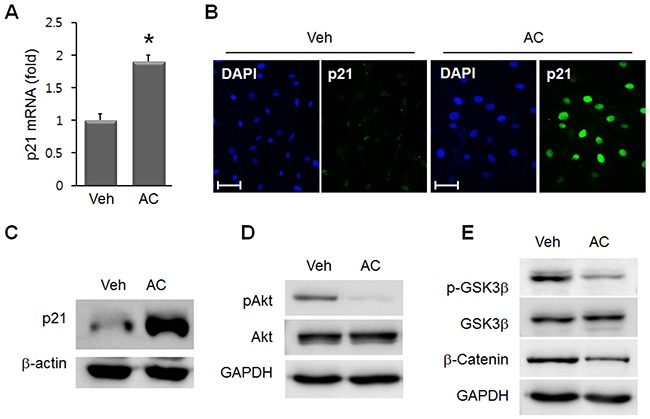
Effects of apicidin on p21, Akt, and GSK3β/β-catenin in MSC **(A)** p21 mRNA was increased in 3 μM AC/MSC. Data are the mean±SEM from at least three independent experiments. *P<0.05. Upregulated p21 protein was assessed by immunofluorescence staining **(B)** and Western blot **(C)** in AC/MSC. **(D)** Phosphorylated Akt was reduced by apicidin treatment. **(E)** Phosphorylated GSK3β and β-catenin were decreased in AC/MSC. Scale bar=50 μm.

In addition to YAP, we examined the cell proliferation-related mediators, p21 and Akt. In AC/MSC, p21, an inhibitory regulator of cell proliferation, was significantly increased at the mRNA (Figure 5A) and protein (Figure 5B, 5C) levels. Phosphorylated Akt, a pro-proliferative mediator, was reduced by apicidin treatment in MSC (Figure 5D). These data indicated the inhibitory effect of apicidin on cell proliferation. Next, the involvement of GSK3β and β-catenin was studied. The protein levels of phosphorylated GSK3β and β-catenin were reduced by apicidin treatment (Figure 5E).

### Downregulation of YAP is necessary for the induction of MSC

**Figure 6:**
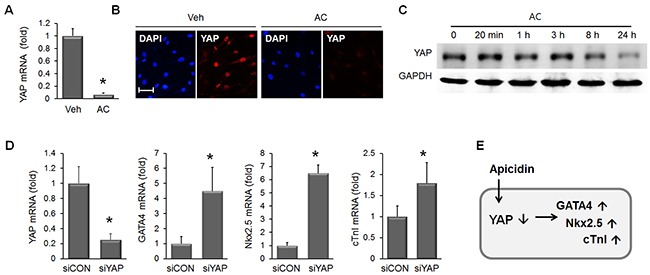
Apicidin-induced YAP downregulation in MSC **(A)** YAP mRNA was significantly downregulated in 3 μM AC/MSC (n=6). **(B)** Immunofluorescence staining showed YAP protein downregulation by apicidin treatment. **(C)** Western blot was performed to validate the YAP protein expression over time course and showed YAP expression gradually decreased between 8h and 24h. **(D)** Knockdown of YAP by RNA interference increased mRNA levels of GATA4, Nkx2.5, and cTnI (n=4). **(E)** Diagram showing apicidin-induced YAP downregulation resulting in the induction of cardiac markers. *P<0.05; Scale bar=50 μm.

Once we confirmed that apicidin could induce cardiogenic induction of MSC, we looked for the responsible mediators. YAP is well known to drive cell proliferation, and we examined the effect of apicidin on YAP in MSC. The mRNA of YAP in MSC was dramatically reduced by apicidin treatment (Figure 6A). YAP protein was also downregulated in AC/MSC as shown by immunocytochemical staining (Figure 6B). Further analysis on the time course Western blot showed the YAP protein began declining at 8h in response to apicidin treatment in MSC (Figure 6C). To examine whether downregulation of YAP was involved in induction of cardiac genes, YAP-knockdown was induced by siRNA transfection. In YAP-knockdown MSC, GATA4, Nkx2.5, and cTnI were upregulated (Figure 6D). The data described above indicated that ablation of YAP by apicidin induces cardiac gene expression in MSC (Figure 6E). The significant reduction of amphiregulin (AREG), a downstream target of YAP, was further confirmed the apicidin-induced YAP suppression (Supplementary Figure 2A). Phosphorylation of YAP was necessary for proteasomal degradation of YAP, but apicidin inhibited YAP phosphorylation (Supplementary Figure 2B). This result showed that the mechanism of YAP downregulation by apicidin was distinct from the classic pathway.

### Apicidin-induced cardiac commitment of MSC involves miR-130a and KLF4

**Figure 7:**
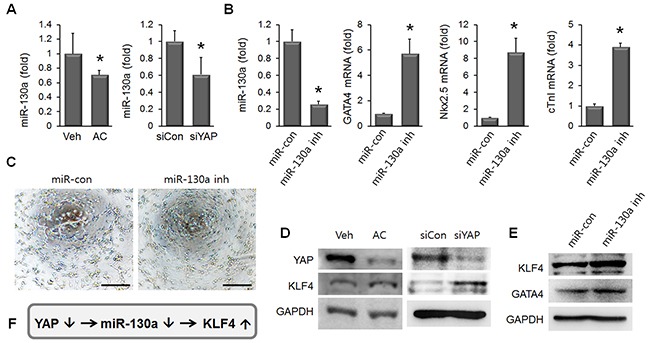
miR-130a and KLF4 are involved in apicidin-induced cardiac commitment of MSC **(A)** miR-130a was reduced by both apicidin treatment and YAP knockdown in MSC (n=4). **(B)** MSC transfected with miR-130a inhibitor showed upregulations of GATA4, Nkx2.5, and cTnI (n=4). **(C)** The representative images showed that tube formation was declined in miR-130a inhibitor-transfected MSC. Scale bar=200 μm. **(D)** KLF4 protein was increased by both apicidin treatment and YAP knockdown. **(E)** miR-130a inhibition induced elevations of KLF4 and GATA4 in MSC. **(F)** Diagram showing that knockdown of YAP induced KLF4 expression via miR-31a downregulation.

To understand the mechanism of YAP suppression-induced cardiac marker inductions in AC/MSC, we came across miR-130a, which was recently reported to be induced by YAP, and also identified as a driver of endothelial differentiation [26, 27]. To test whether miR-130a was involved in apicidin-induced cardiac commitment of MSC, the expression level of miR-130a was assessed in AC/MSC and YAP knockdown MSC. We observed the significant reduction of miR-130a by both apicidin treatment and YAP knockdown in MSC (Figure 7A). To examine whether miR-130a was related with cardiac marker induction in MSC, mRNA levels of cardiac-specific markers was assessed in miR-130a inhibitor-transfected MSC. The levels of mRNA of GATA4, Nkx2.5, and cTnI were markedly increased in miR-130a inhibited MSC (Figure 7B). We showed the tube formation activity of MSC was significantly declined by apicidin treatment (Figure 3A). Tube formation was also decreased in miR-130a reduced MSC (Figure 7C), and this result suggested that apicidin-induced YAP suppression might result in miR-130a reduction. KLF4 is one of the targets of miR-130a [28], and we previously reported that angiogenic activity was declined in KLF4 upregulated MSC [29]. In order to demonstrate the potential linkage of YAP/miR-130a/KLF4 with apicidin-induced cardiac commitment, the expression level of YAP, KLF4, or GATA4 was assessed in MSC after apicidin treatment, YAP knockdown, or miR-130a inhibition. KLF4 protein was elevated along with YAP suppression by apicidin treatment and YAP knockdown in MSC (Figure 7D). Besides, miR-130a-reduced MSC showed the upregulations of both KLF4 and GATA4 (Figure 7E). These results signified that apicidin may determine the cell fate toward cardiac lineage through YAP/miR-130a/KLF4 in MSC (Figure 7F).

## DISCUSSION

Cardiac regeneration therapy is the hottest topic in the medical field. Regenerative cell therapy exerts its therapeutic effects through angiogenesis, anti-apoptosis of neighboring cardiomyocytes, anti-inflammation, and cardiac differentiation to contribute to the replacement of dead cardiomyocytes.

Apart from the paracrine effect of stem cells, the effective induction for cardiomyogenic differentiation of adult stem cells has been hardly achieved despite intense progress in regeneration medicine. For adult stem cells, a number of strategies to induce cardiac differentiation have long been studied. Umbilical cord blood-derived MSC pretreated with oxytocin show increased cardiac gene expression and were successfully incorporated into the myocardium to enhance cardiac recovery [5]. Gap junction formation, cardiomyocyte protection, and the therapeutic efficacy of MSC are enhanced by pretreatment with a growth factor cocktail. Growth factors for induction of cardiac differentiation include bone morphogenetic protein-2 (BMP2), fibroblast growth factor-2 (FGF2), and insulin-like growth factor-1 (IGF1) and the optimized culture period was 7 days [16]. Lineage pre-specification of MSC results in enhanced therapeutic outcomes in patients with chronic ischemic cardiomyopathy. Despite the significant superiority of guided cardiopoiesis in functional recovery, however, it took 4 to 8 weeks to achieve a cardiac phenotype [17].

In this study, immortalized human bone marrow-derived MSC were used, and their differentiation capacities and angiogenic activity have been confirmed in our previous study [29]. We shortened the treatment time of MSC with apicidin to 24 hours, and this treatment protocol was sufficient to induce cardiac genes in MSC. Interestingly, apicidin treatment downregulated the expression of stemness genes and limited the differentiation capacities into osteocytes, chondrocytes, and adipocytes. These orchestrated alterations of AC/MSC might specify cell fate toward the cardiac lineage. In human embryonic carcinoma NCCIT cells, apicidin was shown to downregulate Nanog and enhance the differentiation potential of NCCIT cells to the various lineages [30].

GATA4 is the best studied transcription factor and has multiple distinct roles in cardiac specification, differentiation, morphogenesis, and survival. GATA4 expression is capable of specifying cardiomyocyte fate in embryonic stem cell-derived progenitors [31]. Overexpression of GATA4 results in the upregulation of brain natriuretic peptide, Islet-1, and α-sarcomeric actin in rat bone marrow-derived MSC [32]. In the present study, apicidin-induced GATA4 expression might have driven skewed differentiation to the cardiac lineage in MSC. Interestingly, in AC/MSC, the contractile protein cTnI was located in the nuclei, not in the cytoplasm. This unique distribution was reported previously. cTnI, cTnT, and cTnC were observed in nuclei in cardiomyocyte [33] and cardiac differentiated MSC but not in undifferentiated MSC [34].

YAP is a key downstream molecule of the Hippo signaling pathway that controls cell proliferation and organ size [22]. In stem cells, YAP is crucial to maintain the pluripotency of human and mouse embryonic stem cells, where it acts as a coactivator of the TEAD transcription factors to regulate several stemness genes [23]. The cellular function of YAP within MSC is unclear, however. YAP protein is decreased during embryonic stem cell differentiation. In differentiated embryonic stem cells, GATA4 is increased, whereas Oct4, Sox2, and YAP were reduced [23]. Cell cycle arrest inducer p21 showed a dramatic induction, whereas phosphorylated Akt was significantly reduced in AC/MSC (Figure 5C, 5D). These findings suggested that apicidin treatment was negatively associated with cell proliferation.

GATA4 is a negative regulator of normal astrocyte proliferation and suppresses tumor cell growth through direct activation of p21 [35]. Besides, one of the targets of YAP is p21, and apicidin treatment induces p21 followed by YAP downregulation in MSC. p21 negatively regulates the expression of the pluripotency factor Sox2 in adult neural stem cells [36]. Apicidin may activate and coordinate cardiac commitment programs and may have greater success than regulation of individual genes.

In the present study, phosphorylated GSK3β, an inactive form of GSK3β, and β-catenin were decreased (Figure 5E) in AC/MSC. GSK3β is known to act upstream of the β-catenin-dependent gene regulation machinery, and GSK3β mediates the phosphorylation of β-catenin and subsequently leads to the degradation of β-catenin [37]. In terms of the role of β-catenin in cardiogenesis, downregulation of Nkx2.5 results in β-catenin increase [38]. In other words, an increase in Nkx2.5 by apicidin results in GSK3β-mediated β-catenin degradation.

Distinctly to cardiac commitment of MSC, the impairment of angiogenesis resulting from apicidin treatment may be due to a consequent downregulation of YAP expression. YAP acts as an angiogenic regulator via modulation of angiopoietin-2 expression in endothelial cells. YAP knockdown suppressed tubular network formation of endothelial cells and vascular density was decreased in YAP siRNA-injected mouse retinas [39]. Our study demonstrated that the impairment of angiogenesis resulting from apicidin treatment may be due to a consequent downregulation of YAP expression. Furthermore, miR-130a was identified as one of the downstream effectors of YAP, which resulted in retarded angiogenesis and GATA4 upregulation (Figure 7).

**Figure 8:**
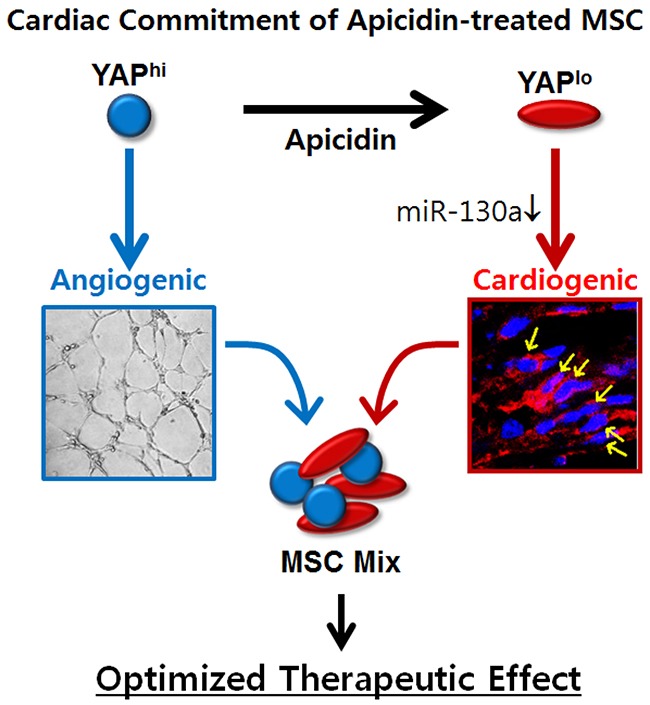
Schematic representation of proposed mechanism of apicidin-mediated MSC fate determination toward cardiac lineage

The cardiac function showed improvement in both the MSC group and the AC/MSC group (Figure 2A). The first time we discovered the effect of apicidin, we highly expected therapeutic efficacy by dramatic inductions of cardiac markers in MSC. However, those significant effects were not reflected in the animal studies. The main mechanisms by which MSC contribute to cardiac regeneration are angiogenesis [4, 29] and the paracrine effect in the infarcted myocardium [40, 41]. Despite better cardiac differentiation of AC/MSC than MSC in the heart (Figure 2B), cardiac fibrosis and angiogenesis showed no improvement (Figure 2C, 2D). From these results, we doubted whether apicidin affected the angiogenesis activity of MSC, which proved to be true. To resolve this unexpected problem, we reformulated our protocol by mixing MSC and AC/MSC. This concept is quite simple and compensates the roles of MSC and AC/MSC to balance cardiac differentiation and angiogenesis. Karantalis *et al*. demonstrated that the coadministration of MSC and cardiac stem cells showed improvement of myocardial contractile performance in pig chronic MI model [42]. They isolated cardiac stem cells from endomyocardial biopsy specimens obtained from the septal wall and coinjected them with MSC. On the other hand, our novel protocol needs MSC alone with or without apicidin priming for 1 day. Both functional angiogenesis and cardiac differentiation were significantly induced by application of the MSC Mix, which was represented as greatly improved cardiac recovery in an animal model (Figure 4). Our results demonstrated that apicidin treatment is clearly involved in cardiogenic gene transcription program and associated with cardiac commitment of MSC, and allow better understanding of the mechanism of cardiac commitment of MSC, although the YAP/miR-130a-related signaling networks remain to be addressed (Figure 8).

To the best of our knowledge, this is the first combined application with the naïve stem cells and fate-committed stem cells to maximize therapeutic efficacy in MI model. Our study suggests that the novel protocol takes advantage of both MSC and AC/MSC.

## MATERIALS AND METHODS

All experiments were approved by the Ethic Committee of Chonnam National University guidelines and regulations. Furthermore, all methods were performed in accordance with guidelines and regulations by the Ethic Committee of Chonnam National University.

### Cell cultures

Human bone marrow-derived MSC immortalized by the introduction of telomerase were kindly provided by Professor Yeon-Soo Kim (Inje University, Inje, South Korea). MSCs were cultured in Dulbecco's Modified Eagle's Medium (DMEM) with 10% fetal bovine serum and 1% penicillin/streptomycin (Gibco, USA). Cells were grown at 37°C in a humidified atmosphere of 95% air and 5% CO_2_, and the medium was changed every 3 days. Apicidin was purchased from Sigma-Aldrich (St Louis, MO, USA).

### Transfection of siRNA and miRNA

RNA interference and miRNA transfection were performed according to the manufacturer's protocols. Briefly, siRNA-control (siCon) and siRNA-specific YAP (siYAP) were purchased from Bioneer (Daejeon, South Korea). MiRNA-control (miR-con) and miRNA-130a were purchased from Dharmacon (GE HearthCare Life Science, USA). Cells were transfected with siRNA or miRNA using Lipofectamine RNAiMAX transfection reagent (Invitrogen, MA, USA). The cells were allowed to grow for another 48 h before being collected for the following experiments.

### Real time-polymerase chain reaction (RT-PCR)

Total RNA from MSC was extracted with TRIzol reagent (Life Technologies, CA, USA), and the RNA samples were converted into complementary DNA by using an Applied Biosystems High-Capacity cDNA Reverse transcription Kit (Invitrogen, MA, USA) according to the manufacturer's instructions. RT-PCR was performed using a QuantiTect SYBR Green PCR kit (Qiagen, Valencia, USA) and Corbett Research Rotor-Gene RG-3000 Real Time PCR System. Pre-designed primers for human GATA4, Nkx2.5, cTnI, Runx2, PPAR-γ, Col2a1, Nanog, Sox2, Oct4, Fog2, Art27, YAP, p21, and AREG were purchased from Bioneer (Daejeon, Korea). MiR-130a was quantified by RT-PCR using TaqMan® MicroRNA Reverse Transcription Kit (Life Technologies, USA). The primers of miR-130a and 18S were purchased from TaqMan® MicroRNA assay (Life Technologies, USA).

### Luciferase reporter assay

GATA4-promoter was kindly provided by Professor Hyun Kook (Chonnam National University Medical School, Gwangju, South Korea). MSCs were plated in 24-well plates and transiently transfected with 1 μg of GATA4 promoter-luciferase reporter and 0.1 μg Renilla luciferase vector using X-tremeGENE HP DNA Transfection Reagent (Roche, Basel, Switzerland). Forty-eight hours later, cells were harvested, and luciferase activity was measured by using the Dual-Luciferase Reporter Assay (Promega, Wisconsin, USA) and a Sirius Luminometer (Berthold, Germany) according to the manufacturer's instructions.

### Flow cytometry

MSC were harvested and washed in wash buffer (PBS containing 1% BSA and 0.1% sodium azide). Cells were fixed with 4% formaldehyde for 10 min and then permeabilized with 0.1% PBS-Tween for 20 min. Cells were then incubated with Alexa 488-conjugated anti-human GATA4 (Abcam, Cambridge, MA, USA) for 30 min. Cells were washed once in wash buffer, and then resuspended in 0.25 mL of 0.5% paraformaldehyde in PBS. Data was acquired by BD FACSCalibur flow cytometer by using CellQuest Pro acquisition software (BD Biosciences, San Jose, CA).

### Western blotting

Whole cell lysates were harvested by using lysis buffer (20 mM Tris-HCl pH 7.4, 0.1 mM EDTA, 150 mM NaCl, 1 mM phenylmethylsulfonyl fluoride and 1 mg/mL leupeptin) on a rotation wheel for 1 h at 4°C. After centrifugation at 10,000 × *g* for 10min, the supernatant was prepared as a protein extract. Equal concentrations of proteins were fractionated by electrophoresis on 8%-12% acrylamide gels and were transferred onto a polyvinylidene fluoride membrane (Merck Millipore, Darmstadt, Germany), followed by blotting with antibodies for GATA4 (Abcam, Cambridge, MA, USA), YAP (Cell Signaling Technology, MA, USA), p21 (Santa Cruz Biotech, Dallas, TX, USA), GSK3β (Santa Cruz Biotech, Dallas, TX, USA), Phospho-GSK3β (Cell Signaling Technology, MA, USA), Akt (Cell Signaling Technology, MA, USA), phospho-Akt (Cell Signaling Technology, MA, USA), β-catenin (Santa Cruz Biotech, Dallas, TX, USA), Krüppel-like factor 4 (KLF4, Abcam, Cambridge, MA, USA), glyceraldehyde 3-phosphoate dehydrogenase (GAPDH, Santa Cruz Biotech, Dallas, TX, USA), and β-actin (Sigma-Aldrich, USA) followed by secondary staining with horseradish peroxidase-conjugated immunoglobulin G. Protein expression was detected by using an Image Reader (LAS-3000 Imaging System, Fuji Photo Film, Tokyo, Japan). The expression level was quantified with Image J (NIH, Bethesda, MD, USA).

### Immunocytochemistry

MSC were fixed in 4% paraformaldehyde for 15 minutes. Then the cell membrane was penetrated by 0.1% Triton X-100 for 10 minutes and blocked by normal goat serum for 1 hour, followed by primary antibody staining at 4°C overnight. Primary antibodies included GATA4 (Abcam, Cambridge, MA, USA), cTnI (Santa Cruz Biotech, Dallas, TX, USA), Nkx2.5 (Santa Cruz Biotech, Dallas, TX, USA), YAP (Cell Signaling Technology, MA, USA), and p21 (Santa Cruz Biotech, Dallas, TX, USA). Subsequently the cells were incubated with secondary antibodies conjugated with Alexa 488 or 594 (Molecular Probes, Invitrogen, CA, USA) for 1 h, followed by mounting with 4,6-diamidino-2-phenylindole (DAPI) (Molecular Probes, Invitrogen, CA, USA).

### *In vitro* angiogenesis assay

Tube formation was assayed by using an *in vitro* angiogenesis assay kit (Merck Millipore, Darmstadt, Germany). Cells (1×10^4^) were plated onto matrix gel-coated 96-well plates and cultured in DMEM without serum. Tube formation was monitored and photographed by using an inverted microscope, and images were analyzed by using Image-Pro software. Angiogenic activity was quantified by measuring tube length. Total tube length in four fields per well was averaged.

### Matrigel plug assay

PBS or MSC (2×10^6^ cells) were mixed with phenol-red-free Matrigel (Matrigel™ Basement Membrane Matrix High Concentration, BD Biosciences, USA) and subcutaneously injected into 8-week-old male Balb/c athymic nude mice. After 14 days, the Matrigel plugs were harvested and processed for analysis. To estimate the degree of vascularization, H&E-stained digital images were analyzed by measuring the erythrocyte-filled area and expressing that as a percentage of the total area of Matrigel.

### Myocardial infarction and cell transplantation in a mouse model

Male inbred Balb/C nude mice (7-8 weeks of age) were purchased from ORIENT BIO Inc. (Korea). This study was reviewed and approved by the Chonnam National University Institutional Animal Care and Use Committee (CNU IACUC-H-2014-21), and all experiments were performed after approval by our local ethical committee at Chonnam National University Medical School. Mice were anesthetized with an intramuscular injection of ketamine (50 mg/kg) and xylazine (5 mg/kg), intubated, and mechanically ventilated. The proximal left anterior descending coronary artery was ligated. After 7 days, the mice were randomly divided into 4 groups (n=10 each group) and anesthetized for reoperation. For transplantation into mouse hearts, MSC (3×10^5^ diluted in 30 μL of PBS) labeled with DAPI for 1 day were prepared. MSC were treated with vehicle or apicidin (3 μM) for 1 day before transplantation. Mice were injected with PBS alone, MSC, AC/MSC or MSC+AC/MSC (MSC Mix) into the peri-infarct area of left ventricular myocardium. For the MSC Mix group, MSC (1.5×10^5^) and AC/MSC (1.5×10^5^) were mixed before cell injection. At 2 weeks after MSC injection, the mice were sacrificed and the hearts rapidly were harvested for further analysis.

### Echocardiography

Cardiac function was assessed by echocardiography. At 2 weeks after injection of MSC, the mice were anesthetized, intubated, and mechanically ventilated. Their cardiac function, including EF and fractional shortening, was measured by transthoracic echocardiography (15-MHz linear array transducer system; iE33 system, Philips Medical Systems; Amsterdam, Netherland). Fractional shortening was calculated as 100×(LVDd-LVDs)/LVDd (%).

### Histological analyses and immunohistochemistry

For immunohistochemical analysis, the hearts were harvested and embedded in Tissue Tek O.C.T. compound (Leica, Germany) and frozen in liquid nitrogen. Frozen tissues were cut at a thickness of 10 μm and were mounted on glass slides for staining. Frozen slides were stained with primary antibodies against cTnI (Santa Cruz Biotech, Dallas, TX, USA), von Willebrand Factor (vWF; Sigma-Aldrich, MO, USA) and α-smooth muscle actin (α-SMA; Sigma-Aldrich, MO, USA). The images were detected by using a Carl Zeiss confocal microscope, and the images were obtained using Zeiss LSM version 3.2 SP2 software (Carl Zeiss, Germany). Cardiac fibrosis was measured by Masson's Trichrome staining, and fibrotic areas were measured by visualizing blue-stained fibrotic deposits by using NIS-Elements.

### Statistical analysis

All data are expressed as the mean±SEM from at least three independent experiments. The differences between experimental and control groups were analyzed with the two-tailed unpaired Student's *t*-test by using SPSS (SPSS Inc., Chicago, IL, USA). A value of *P*<0.05 was considered statistically significant.

## SUPPLEMENTARY MATERIALS FIGURES AND TABLES


