# Supplementary material for: The optimization of cell therapy by combinational application with apicidin-treated mesenchymal stem cells after myocardial infarction
Source: Oncotarget. 2017 Apr 27;8(27):44281–94. doi: 10.18632/oncotarget.17471 (PMC5546480; doi:10.18632/oncotarget.17471)
Supplement: Supplementary file 1 [file oncotarget-08-44281-s001.pdf]

The optimization of cell therapy by combinational application with apicidin-treated mesenchymal stem cells after myocardial infarction

SUPPLEMENTARY MATERIALS

Supplementary Methods

Materials

Antibodies against acetylated histone H3 (AcH3, Merck Millipore, Darmstadt, Germany), acetylated histone H4 (AcH4, Merck Millipore, Darmstadt, Germany), phospho-YAP (Ser397) (Cell Signaling Technology, MA, USA), and phospho-YAP (Ser127) (Cell Signaling Technology, MA, USA) were purchased.

HDAC activity assay

HDAC activity for effects of HDAC inhibitors was measured in MSC using the HDAC-Glo I/II Screening Assay (Promega, Wisconsin, USA). In brief cell supernatants were mixed in HDAC-Glo I/II reagent assay mix, containing the substrate and the developer reagent, and transferred to 96-well plates and after 30 min incubation at room temperature, luminescence was measured.

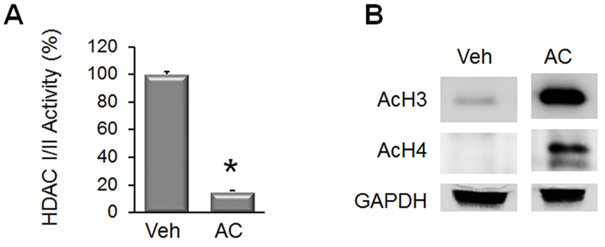

Supplementary Figure 1: Histone deacetylase (HDAC) activity was assessed in MSC treated with vehicle (Veh) or apicidin (AC, 3 µM) for 24 hours. (A) Whole HDAC activity was inhibited in HDAC inhibitor-treated MSCs. **(B)** Acetylated histone 3 (AcH3) and acetylated histone 4 (AcH4) were dramatically increased in apicidin-treated MSC.

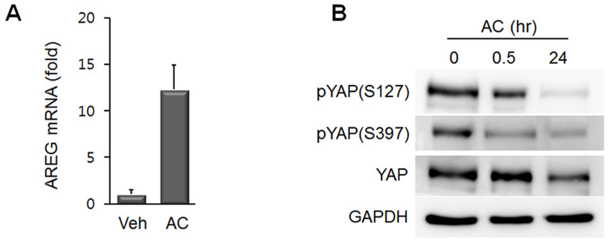

Supplementary Figure 2: Apicidin treatment decreased YAP. (A) mRNA of AREG, a downstream target of YAP, was increased in apicidin-treated MSC. **(B)** Phosphorylation of YAP protein was also reduced by apicidin treatment along with YAP protein downregulation.

Supplementary Table 1: Echocardiographic evaluation after myocardial infarction (MI)

	MI + PBS	MI + MSC	MI + AC/MSC
IVSd (mm)	0.512 ± 0.059	0.555 ± 0.085	0.490 ± 0.077#
IVSs (mm)	0.585 ± 0.108	0.571 ± 0.010	0.571 ± 0.110
LVIDd (mm)	4.331 ± 0.859	4.107 ± 0.054	4.195 ± 0.435
LVIDs (mm)	3.565 ± 0.256	3.200 ± 0.424*	3.143 ± 0.349*
LVPWd (mm)	0.546 ± 0.090	0.543 ±0.051	0.505 ± 0.086
LVPWs (mm)	0.734 ± 0.127	0.657 ± 0.134*	0.624 ± 0.148*
EDV (mL)	0.221 ± 0.046	0.191 ± 0.072	0.187 ± 0.056*
ESV (mL)	0.119 ± 0.028	0.088 ± 0.032*	0.081 ± 0.026*
EF (%)	47.196 ± 2.878	53.995 ± 3.372*	56.427 ± 1.797*#
SV (mL)	0.105 ± 0.022	0.104 ± 0.040	0.108 ± 0.030
FS (%)	20.055 ± 1.531	23.761 ± 0.040*	25.148 ± 1.035*#

MSC, mesenchymal stem cells; AC/MSC, apicidin-treated MSCs, IVSd, intraventricular septal width in diastole; IVSs, intraventricular septal width in systole; LVIDd, left ventricular internal dimension in diastole; LVIDs, left ventricular internal dimension in systole; LVPWd, left ventricular posterior wall thickness in diastole; LVPWs, left ventricular posterior wall thickness in systole; EDV, end-diastolic volume; ESV, end-systolic volume; EF, ejection fraction; SV, stroke volume; FS, fractional shortening

*; $p < 0.05$ vs. MI+PBS group, #; $p < 0.05$ vs. MI+MSC group

Supplementary Table 2: Echocardiographic evaluation after myocardial infarction (MI)

	MI + AC/MSC	MI + MSC Mix
IVSd (mm)	0.370 ± 0.048	0.333 ± 0.049
IVSs (mm)	0.340 ± 0.070	0.333 ± 0.065
LVIDd (mm)	3.690 ± 0.507	4.133 ± 0.644
LVIDs (mm)	3.040 ± 0.458	3.183 ± 0.603
LVPWd (mm)	0.444 ± 0.073	0.408 ±0.051
LVPWs (mm)	0.478 ± 0.083	0.467 ± 0.078
EDV (mL)	0.132 ± 0.053	0.185 ± 0.085
ESV (mL)	0.078 ± 0.040	0.089 ± 0.049
EF (%)	43.508 ± 4.765	52.863 ± 5.780*
SV (mL)	0.061 ± 0.018	0.100 ± 0.036*
FS (%)	18.051 ± 2. 316	23.169 ± 3.175*

MSC, mesenchymal stem cells; AC/MSC, apicidin-treated MSCs, IVSd, intraventricular septal width in diastole; IVSs, intraventricular septal width in systole; LVIDd, left ventricular internal dimension in diastole; LVIDs, left ventricular internal dimension in systole; LVPWd, left ventricular posterior wall thickness in diastole; LVPWs, left ventricular posterior wall thickness in systole; EDV, end-diastolic volume; ESV, end-systolic volume; EF, ejection fraction; SV, stroke volume; FS, fractional shortening *; $p < 0.05$ vs. MI+PBS group

*; $p < 0.05$ vs. MI+PBS group
